# A brain-based pain facilitation mechanism contributes to painful diabetic polyneuropathy

**DOI:** 10.1093/brain/awx337

**Published:** 2018-01-15

**Authors:** Andrew R Segerdahl, Andreas C Themistocleous, Dean Fido, David L Bennett, Irene Tracey

**Affiliations:** 1 Wellcome Centre for Integrative Neuroimaging, FMRIB, Nuffield Department of Clinical Neurosciences, University of Oxford, Oxford, UK; 2 Nuffield Department of Clinical Neurosciences, University of Oxford, Oxford, UK

**Keywords:** neuropathic pain, brainstem, functional connectivity, diabetic neuropathy, facilitation

## Abstract

The descending pain modulatory system represents one of the oldest and most fundamentally important neurophysiological mechanisms relevant to pain. Extensive work in animals and humans has shown how a functional imbalance between the facilitatory and inhibitory components is linked to exacerbation and maintenance of persistent pain states. Forward translation of these findings into clinical populations is needed to verify the relevance of this imbalance. Diabetic polyneuropathy is one of the most common causes of chronic neuropathic pain; however, the reason why ∼25–30% of patients with diabetes develop pain is not known. The current study used a multimodal clinical neuroimaging approach to interrogate whether the sensory phenotype of painful diabetic polyneuropathy involves altered function of the ventrolateral periaqueductal grey—a key node of the descending pain modulatory system. We found that ventrolateral periaqueductal grey functional connectivity is altered in patients suffering from painful diabetic polyneuropathy; the magnitude of which is correlated to their spontaneous and allodynic pain as well as the magnitude of the cortical response elicited by an experimental tonic heat paradigm. We posit that ventrolateral periaqueductal grey-mediated descending pain modulatory system dysfunction may reflect a brain-based pain facilitation mechanism contributing to painful diabetic polyneuropathy.

## Teaser

Diabetic polyneuropathy is a leading cause of chronic neuropathic pain, but the mechanism underlying this link is unknown. Using a multimodal neuroimaging approach, Segerdahl *et al.* show dysfunction of the descending pain modulatory system in those patients with neuropathic pain, which is associated with amplified brain activity in response to painful stimuli.

## Introduction

Chronic pain is one of the largest and costliest medical health problems in the developed world and it is likely to worsen (Gaskin and Richard, 2012; Fayaz *et al.*, 2016). Diabetic polyneuropathy (DPN) is a major factor contributing to this increase: the global prevalence of diabetes mellitus is inexorably rising, 30–50% of such patients develop DPN and up to 50% of these will develop neuropathic pain, which is currently inadequately treated (Feldman *et al.*, 2017).

We need to interrogate what the underlying neurophysiological mechanisms are that precipitate and maintain chronic pain (Tracey, 2017). Extensive work in preclinical animal models highlights the importance of the descending pain modulatory system (DPMS) in chronic pain states (DeFelice *et al.*, 2011; Wang *et al.*, 2013). The DPMS is defined as a brainstem–subcortical–cortical network that can modulate nociceptive input to the brain—either by amplifying or attenuating afferent nociceptive input at the dorsal horn of the spinal cord (Basbaum and Fields, 1978; Fields, 1992; Gebhart, 2004; Mason, 2012). As such, the functional balance between inhibitory and facilitatory arms of this network powerfully controls the resultant afferent nociceptive volley to the brain and hence the resultant pain experience. A primary hypothesis that has emerged from this literature is that persistent pain is linked to an imbalance in DPMS function—either due to a diminished inhibitory and/or an enhanced facilitatory capacity of the DPMS (Keay and Bandler, 2002; Lumb *et al.*, 2002, 2004; Porreca *et al.*, 2002; Suzuki *et al.*, 2004; Heinricher *et al.*, 2009; DeFelice *et al.*, 2011; Wang *et al.*, 2013).

In humans, neuroimaging studies of central sensitization in either experimental medicine models or patients confirm that key nuclei of the DPMS play fundamental roles in the maintenance of central sensitization pain, mechanical hyperalgesia and mechanical allodynia (Iannetti *et al.*, 2005; Zambreanu *et al.*, 2005; Mainero *et al.*, 2007; Lee *et al.*, 2008).

Forward translation of these findings into different patient groups is starting to emerge, as is evidence supporting the possible role of brainstem-mediated facilitation underlying different features of clinical pain (Gwilym *et al.*, 2009; Schwedt *et al.*, 2014; Yu *et al.*, 2014; Soni *et al.*, 2016; Truini *et al.*, 2016).

While these studies have shown a link between DPMS dysfunction and clinical measures of pain, the precise mechanism by which the DPMS contributes to pain facilitation in chronic pain is unclear.

We hypothesized that the sensory phenotype of DPN patients with pain (NP+) involves altered connectivity of the ventrolateral periacqueductal grey (vlPAG)—a vital nucleus within the DPMS—to key pain processing brain regions. Further, that the extent to which it is connected is related to key features of their pain: namely, the intensity of their spontaneous background pain and their brain response and pain intensity rating to a tonic heat hyperalgesia challenge. We interrogated this in DPN patients who were meticulously well matched for key clinical markers of disease progression but within whom only a percentage suffer from persistent neuropathic pain (Themistocleous *et al.*, 2016). We posit that the altered vlPAG functional connectivity profile in NP+ patients may reflect a brain-based pain facilitation mechanism underlying this notoriously debilitating disease.

## Materials and methods

### Participants

Thirty individuals were recruited from the Pain in Neuropathy Study (PiNS, approved by the National Research Ethics Service, UK: No: 10/H07056/35), and written informed consent was obtained according to the Declaration of Helsinki. Refer to Themistocleous *et al.* (2016) for a detailed description of PiNS. Data from two participants were excluded because of scanner-related problems and a further two participants were excluded because of illness during scanning.

### Sensory phenotyping

The participants were studied intensively using a range of clinical, neuroimaging and behavioural tests. Basic clinical parameters were measured for each participant (weight, height, and blood pressure) (Supplementary Table 1). A structured neurological examination including assessment of nerve conduction, skin biopsy and quantitative sensory testing (QST) were completed. Additional drug, laboratory, and clinical investigation data from the clinical records were also collected. All study participants had diabetes mellitus with evidence of clinical length-dependant neuropathy confirmed by abnormalities on either nerve conduction studies or intra-epidermal nerve fibre density (IEFND) (Tesfaye *et al.*, 2011). The presence of a diabetic peripheral neuropathy was confirmed using the comprehensive upper and lower limb neurological examination, nerve conduction testing, determination of IEFND from skin biopsies, and the battery of QST measures. Further details can be found in the Supplementary material and in Themistocleous *et al.* (2016). All participants met the American or the Toronto consensus criteria for a definite diabetic peripheral neuropathy.

### Painful versus painless diabetic polyneuropathy

The presence of chronic neuropathic pain caused by peripheral DPN was determined at the time of the clinical assessment and was in line with the International Association for the Study of Pain (IASP) definition of neuropathic pain i.e. ‘pain caused by a lesion or disease of the somatosensory system’. The IASP/NeuPSIG grading system was used to grade the neuropathic pain (Finnerup *et al.,* 2016). Participants were divided into those with NP+ (painful diabetic neuropathy) and those without, non-NP (painless diabetic neuropathy). Only study participants with chronic painful diabetic neuropathy present for at least 3 months were included in the NP+ group. Study participants with non-neuropathic pain in the extremities, such as musculoskeletal pain of the ankle, were included in the non-NP group. A battery of questionnaires was used to quantify and monitor features of the NP+ pain; including: the 7-day pain intensity diary, Douleur Neuropathique en 4 Questions (DN4); PainDETECT; Brief Pain Inventory (BPI); and the Neuropathic Pain Symptom Inventory (NPSI). A detailed description of the questionnaires can be found in the Supplementary material and in Themistocleous *et al.* (2016).

SPSS Statistics Version 21 (IBM), GraphPad Prism, and JASP were used for all statistical analyses. QST z-score data were expressed as mean ± 95% confidence interval (CI). All other data were not normally distributed and reported as the median with interquartile range (IQR). QST z-scores were compared with unpaired *t*-tests and all other data with Mann-Whitney U-tests. Categorical data were analysed with Fischer’s exact test. Significance was set at *P* = 0.05.

### Functional MRI

All participants were scanned using a Siemens 3 T Verio whole-body magnetic resonance scanner equipped with a 32-channel head and a body coil. T_1_-weighted structural images were acquired with a 3D MPRAGE sequence (1 × 1 × 1 mm voxels). Five minutes of T_2_*-weighted blood oxygen level-dependent (BOLD) ‘resting state’ data were acquired using a multi-band (version 6) sequence (repetition time = 1.3 s; echo time = 40 ms; 2 × 2 × 2 mm voxels; 72 slices). Subjects were asked to remain still with their gaze focused on a stationary visual cue. Absolute cerebral blood flow (CBF) data were acquired using a multi-inversion time pseudo-continuous arterial spin labelling (*p*CASL) sequence described previously (Segerdahl *et al.*, 2015). A total of 114 volumes were acquired (∼7 min of scan time) for each condition. A full description of the sequence parameters used are in the Supplementary material. Briefly, ‘tag’ and ‘control’ images were acquired every repetition time = 4 s with a label duration of 1.4 s. A total of six inversion times were used. B_0_ shimming was performed over the imaging region and the labelling plane to minimize off-resonance effects. All participants were scanned at rest (7 min) and then during the experience of tonic heating of the subject’s feet using a temperature controlled water bottle (temperature = 44°C; as measured throughout scans using an infrared thermometer). Participants verbally rated the intensity of their background pain at rest and during the tonic heat stimulation paradigm using a 0–10 scale. Ratings were collected immediately preceding the start and following the end of each scan. A schematic of the scan paradigm is displayed in Fig. 2A.

### Functional MRI statistical analysis

MRI data acquisition, preprocessing and analyses followed standard procedures (Smith *et al.*, 2004). All functional MRI analysis was completed in each subject’s native anatomical space and then was co-registered to a standard MNI152 template brain using non-linear registration (FNIRT).

BOLD functional data were preprocessed using FSL FMRIB’s expert analysis tool (FEAT, version 6.0), using a kernel of full-width at half-maximum of 2 mm for spatial smoothing. Voxel-wise seed-based functional connectivity analyses were completed using standard methods (Fox *et al.*, 2006). The seed was defined by a 7 T anatomical mask for the vlPAG published previously (Ezra *et al.*, 2015; Faull and Pattinson, 2017); warped to standard 3 T space (2 mm isotropic) using FNIRT and then visually inspected for accuracy. Nuisance regions of interest for CSF and white matter were generated for each subject. The first Eigen time series of the seed and regions of interest were calculated; which is the single time series that best reflects coherent activity across the mask by reflecting the largest amount of total variance that constitutes that mask. Conditional correlations between the seed and each voxel in each subject’s brain volume were calculated by regressing out the Eigen time series of each nuisance region of interest. This produces a set of partial correlation coefficients for the seed relative to the whole brain that are not shared by the nuisance regions of interest. The conditional correlation coefficients for each subject’s seed were entered into an unpaired *t*-test at the group level alongside a regressor for all subjects’ background pain ratings (mixed effects; z > 3.1, *P* < 0.05).

ASL functional data were preprocessed using previously published methods that adhere to current guidelines (Alsop *et al.*, 2015; Segerdahl *et al.*, 2015). Refer to the Supplementary material for more details. The absolute CBF time series generated for each subject during rest and during tonic heating was averaged using a mixed effects model (to account for voxel-wise variance of the Bayesian fit during CBF quantification). This generated a single whole brain voxel-wise absolute CBF volume for each subject at each condition with a corresponding variance image for use at the group level. Each subject’s whole brain absolute CBF volume during ‘rest’ and ‘heat’ conditions were used at the group level to determine the group difference (NP+ > non-NP) in heat-evoked perfusion as a function of each subject’s resting vlPAG functional connectivity strength with the rostral anterior cingulate cortex (rACC) as observed with the BOLD sequence (Fig. 4A: mixed effects; z > 3.1, *P* < 0.05). The rACC was chosen because it was observed to have the peak maximum functional connectivity strength with the vlPAG (Fig. 2), it has connections with the PAG, and top-down facilitation of pain via this pathway has been shown previously in rodents (Calejesan *et al.*, 2000; Porreca *et al.*, 2002). Additionally, a linear correlation between the vlPAG functional connectivity strength and the tonic-heat evoked pain intensity ratings was tested for each group.

## Results

### Demographics, diabetic polyneuropathy sensory phenotype and pain severity

**Figure 1:**
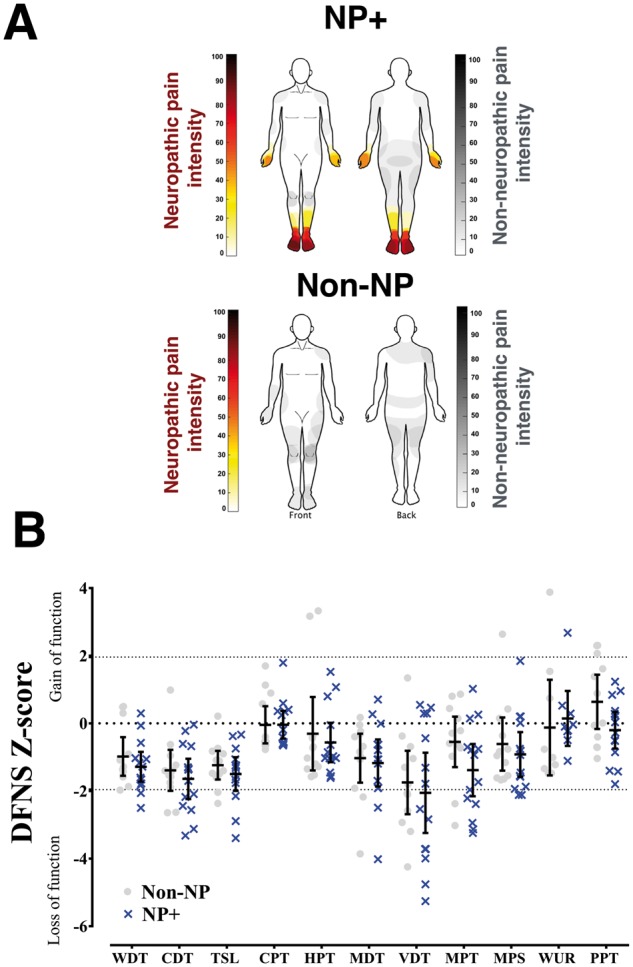
**Neuropathic pain severity*.*** (**A**) Heat maps summarize the mean pain intensity and pain location on the body as generated from each participant’s 7-day pain diary. Diabetes-associated neuropathic pain is shown in red and non-neuropathic pain is in grey for both groups. (**B**) Scatter plot and mean ± 95% CI of z-scores for QST parameters for each group (NP+: blue; non-NP: grey). The z-score indicates the number of standard deviations the participant data are from the mean of the control population (i.e. the normative data). A z-score that lies between −2 and +2 is considered within the normal reference range. Positive z-scores denote gain of function, whereas negative z-scores denote loss of function. CDT = cold detection threshold; CPT = cold pain threshold; HPT = heat pain threshold; MDT = mechanical detection threshold; MPS = mechanical pain sensitivity; MPT = mechanical pain threshold; PPT = pressure pain threshold; TSL = thermal sensory limen; VDT = vibration detection threshold; WDT = warm detection threshold; WUR = wind-up ratio.

**Figure 2:**
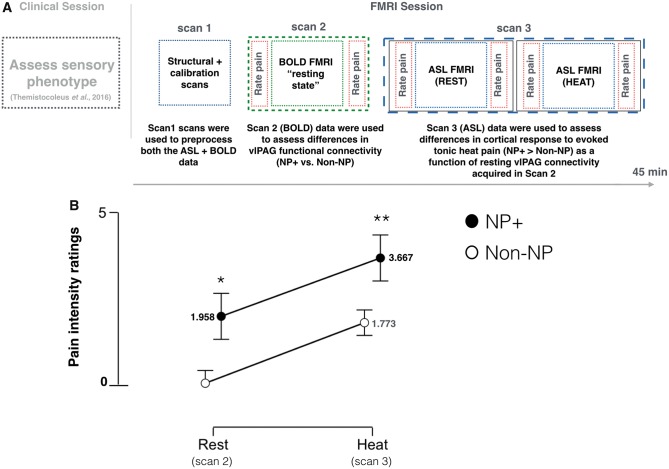
**Experimental pain psychophysics.** (**A**) A schematic summarizing the experimental design of the study. Patients’ sensory phenotype were confirmed during a clinical examination, which occurred on a separate day that preceded the functional MRI session. The functional MRI session consisted of three different scans: Scan 1 = high-resolution structural and calibration scans; Scan 2 = BOLD functional MRI resting state data were acquired; Scan 3 = ASL functional MRI data were acquired during evoked tonic heat applied to the subjects’ feet. Each scan is represented visually by a different colour. Verbal pain intensity ratings were obtained immediately before and after each scan using the 11-point numerical rating scale (0 = no pain; 10 = worst pain imaginable). Ratings were collected between scan data collection (i.e. the scanner was ‘off’ during this time). (**B**) The comparison of the group mean pain intensity ratings collected at rest (i.e. Scan 2) and during tonic heating (i.e. Scan 3) between NP+ and non-NP participants (Mann-Whitney U-test, Rest: *P = 0.001; Heat: **P = 0.025). Error bars represent the standard deviation of the mean. FMRI = functional MRI.

No significant differences in age, diabetic control (HbA1c), gender, ethnicity, body mass index, and waist-hip circumference were observed between the groups (Fischer’s exact test; *P < 0.05; Supplementary Table 1). Figure 1A shows the distribution and mean intensity (0–100 scale) of the pain associated with NP+ (red) and non-NP (grey) using the 7-day pain diary. Both groups were well matched across almost all QST parameters tested and no significant differences were observed between the groups (Fig. 1B). The NP+ participants scored significantly higher on both the neuropathic and psychological screening tools (Mann-Whitney U-tests, *P* < 0.05; Supplementary material). The group mean pain intensity ratings at rest (Scan 2) and during tonic heat stimulation (Scan 3) are shown in Fig. 2B. NP+ participants reported significantly greater pain for both conditions (Mann-Whitney U-test; Rest: *P* = 0.001; Heat: *P* = 0.025).

### Enhanced ventrolateral periaqueductal grey functional connectivity in NP+ versus non-NP

**Figure 3:**
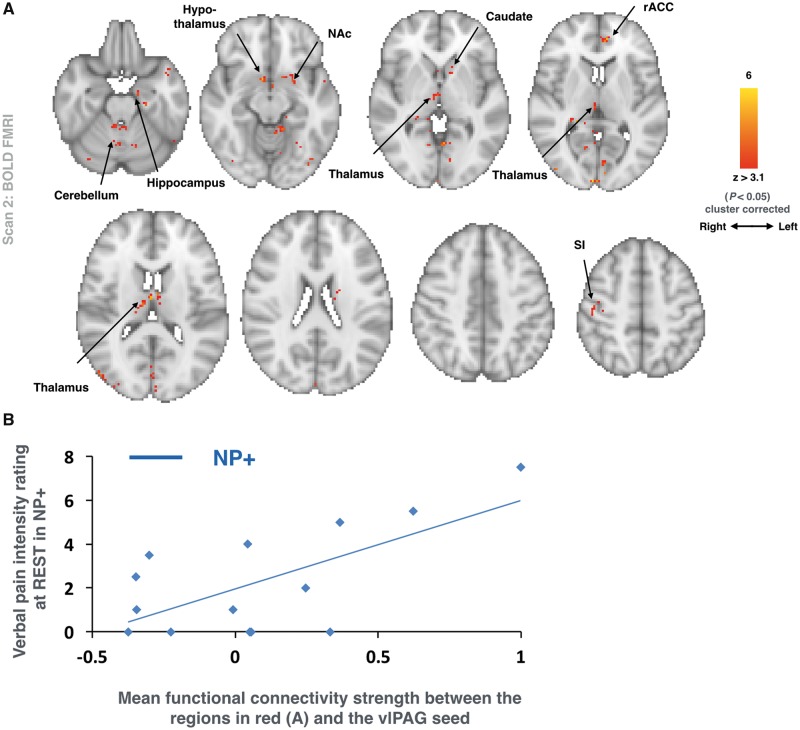
**Enhanced vlPAG functional connectivity in NP+.** (**A**) Group comparison (NP+ > Non-NP) of whole brain functional connectivity strength with the vlPAG seed (from Scan 2) as a function of the background (i.e. ‘resting’) pain intensity ratings (from Scan 2). Voxels within which this relationship was greater in the NP+ group are in red (*n* = 26; mixed effects: z > 3.1, *P* < 0.05 cluster corrected). The axial slices show the extent of activation across the whole brain. Radiological convention is used. (**B**) For clarity, a plot of the correlation between the magnitude of vlPAG functional connectivity and the intensity of the pain at rest reported by NP+ participants is displayed.

There was a significant interaction between pain intensity ratings at rest and the vlPAG functional connectivity with the whole brain between the two groups (Fig. 3A; mixed effects; z > 3.1, *P* < 0.05 cluster corrected). Regions that showed an enhanced connectivity with the seed as a correlate of the intensity of the background spontaneous pain reported included: bilateral thalamus and cerebellum; the right hypothalamus and primary somatosensory cortex (SI); and the left amygdala, nucleus accumbens (NAc), caudate and rACC.

### Ventrolateral periaqueductal grey connectivity predicts heat hyperalgesia

**Figure 4:**
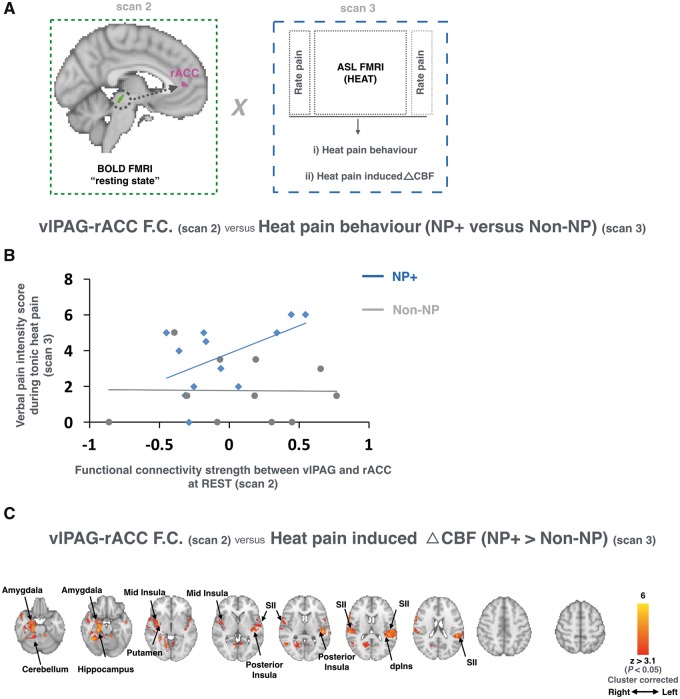
**vlPAG functional connectivity predicts heat hyperalgesia.** (**A**) A schematic of the analysis workflow used to test the relationship between the functional connectivity (F.C.) strength of the vlPAG-rACC (acquired in Scan 2) and the patients’ behavioural (**B**) and brain (**C**) responses to evoked tonic heat applied to their feet (acquired in Scan 3). (**B**) A linear correlation between resting vlPAG-rACC functional connectivity strength (from Scan 2) and the intensity of the pain reported during evoked tonic heat applied to the participants’ feet (from Scan 3; NP+: blue; non-NP: grey). (**C**) Tonic-heat induced hyper-perfusion (CBF; acquired in Scan 3) as a function of resting vlPAG-rACC functional connectivity (acquired during Scan 2). Regions within which the correlation between tonic heat induced changes in CBF and vlPAG-rACC connectivity strength were greater in NP+ versus non-NP patients are shown in red (*n* = 26, mixed effects, z > 3.1, *P* < 0.05 cluster corrected). The axial slices show the extent of activation across the whole brain. Radiological convention is used. FMRI = functional MRI.

Figure 4B plots the correlation between the verbal pain intensity ratings during tonic heat stimulation and the strength of the vlPAG-rACC functional connectivity pre-tonic heat stimulation for both NP+ (blue) and non-NP (grey) patients. In NP+ patients, this correlation was positive (r = 0.531, *P* = 0.038); while in non-NP patients no relationship was observed (r = 0.228; *P* = 0.50). Further, a direct comparison between the groups shows this difference in correlation is significant (Fischer r-to-z transformation; *P < 0.01). Regions within which the correlation between the tonic heat-induced hyperperfusion and the strength of the resting vlPAG–rACC functional connectivity was greater in NP+ versus non-NP patients is displayed in Fig. 4C (mixed effects; z > 3.1, *P* < 0.05; cluster corrected). Regions showing this effect include: bilateral secondary somatosensory cortex (SII), posterior insula and cerebellum; the left dorsal posterior insula (dpIns); and the right hippocampus, mid-insula and amygdala.

## Discussion

Using a multi-modal imaging approach, these data show that the vlPAG functional connectivity is altered in NP+ versus non-NP DPN patients; and that the extent to which it is altered is linked to different features of their pain behaviour and the magnitude of the cortical response elicited by an experimental tonic heat paradigm. While the link between the vlPAG connectivity strength and the patient’s pain behaviour offers support to the idea that there is PAG-mediated amplification of the NP+ patients pain, an essential further line of evidence for a brain-based facilitation mechanism comes from the difference in tonic heat-evoked CBF responses between the two groups. These data show that the vlPAG-rACC connectivity strength is also positively correlated with the magnitude of the tonic heat-induced CBF response in the NP+ > non-NP patients. Thus, only in the NP+ patient group is the altered vlPAG function linked to both the behavioural and brain responses to heat hyperalgesia.

A key feature of the DPMS is that it is possible to modify the magnitude of nociceptive input arriving at the cortex at the level the dorsal horn of the spinal cord; consequently, an alteration in this capacity may lead to a change in the pain experienced simply because the nociceptive barrage arriving in the brain is different. Because the current study did not have access to a reliable simultaneous spinal-brain functional MRI method, we don’t know if there are differences between the groups in the net nociceptive input from the periphery to the spinal cord then on to the brain even though the temperature applied to the participants’ feet was the same. However, it is possible that the positive correlation between the vlPAG connectivity and the enhanced heat-induced CBF response of regions like the insula, including the dorsal posterior insula (dpIns), and SII means that the heat hyperalgesia in NP+ patients is related to vlPAG mediated facilitation of incoming heat input; and that cortical regions, which are known to track key features of thermal input in healthy controls retain a similar function in these patients (Coghill *et al.*, 1999; Craig *et al.*, 2000; Segerdahl *et al.*, 2015).

The current study provides novel insight into the multiple ways in which the DPMS is implicated in painful DPN. While previous work shows how DPN patients have a diminished capacity for descending inhibition, these data provide evidence for a parallel mechanism to be involved that may include the vlPAG, working in conjunction with key nodes of the DPMS (e.g. rACC, amygdala, and hypothalamus) to amplify pain in NP+ patients. Future work employing combined spinal-brain functional MRI will make it possible to interrogate how these parallel processes are related, the extent to which variability in this system may be a useful predictor for the development of different types of chronic pain and possibly, the extent to which this kind of brain-based facilitation mechanism is reversible with treatment.

## Supplementary Material

Supplementary Tables

Supplementary Data

## Abbreviations

DPMS: descending pain modulatory system
DPN: diabetic polyneuropathy
NP+: painful diabetic neuropathy
non-NP: painless diabetic neuropathy
rACC: rostral anterior cingulate cortex
vlPAG: ventrolateral periaqueductal grey
