# Supplementary material for: A brain-based pain facilitation mechanism contributes to painful diabetic polyneuropathy
Source: Brain. 2018 Jan 15;141(2):357–64. doi: 10.1093/brain/awx337 (PMC5837628; doi:10.1093/brain/awx337)
Supplement: Supplementary Tables [file brain-2017-01107-file006_awx337.pdf]

	Painful diabetic neuropathy	Painless diabetic neuropathy	P
**Number of participants**	14	12	
**Age, y**	60.5 (54.2-71.9)	63.5 (57.8-73)	0.44
**Male**	11 (78.6%)	9 (75%)	0.83
**HbA1c, %**	8.3 (7.2-9.3)	6.9 (6.3-8.4)	0.09
**[mmol/mol]**	[67 (55-76)]	[52 (45-68)]	
**Type 2 diabetes**	13 (93%)	12 (100%)	0.99
**Duration of diabetes, y**	10 (8.4 -14.6)	13.9 (10.6 -21.1)	0.08
**BMI, kg/m^2**	29.2 (27.5 -34.4)	30.5 (27.6-34.4)	0.59
**Waist-hip circumference ratio**	0.99 (0.94-1.03)	0.96 (0.88-1.07)	0.46
**Ethnicity**			
**Caucasian**	14 (100%)	12 (100%)	0.99

Supplementary table 1

A summary of the key demographic details and blood results of the participants that were scanned in the study (Mann-Whitney U Test; *$p<0.05$). Data is shown as the median (intra-quartile range).

	Painful diabetic neuropathy	Painless diabetic neuropathy	P
Number of participants	14	12	
**Analgesic use**			
Participants reported analgesic drug use	7	2	0.11
Paracetamol	3	0	0.22
NSAIDS	4	1	0.33
Antidepressants			
Tricyclic Antidepressants (amitriptyline, nortriptyline, imipramine)	2	1	0.99
SNRI (duloxetine)	1	0	0.99
Gabapentanoids			
Gabapentin	2	0	0.48
Pregabalin	2	1	0.99
Topical lidocaine patches	0	0	
Tramadol	0	0	
Opioids			
Weak (codeine)	1	0	0.99
Strong (fentanyl patches, oral morphine)	0	0	
Reported drug use	14	12	
All oral hypoglycaemic agents			
Metformin	12	11	0.99
Other Oral hypoglycaemic (Gliclazide)	6	7	0.70
Insulin	6	5	0.99
Other agents (Sitagliptin, Pioglitazone, Exanatide, Linagliptin)	3	4	0.70
Statin	14	7	**< 0.05**
Aspirin	2	6	0.09
Anti-Hypertensives			
ACE-I/ARB	5	11	**<0.01**
Diuretic	1	1	0.99
B-blocker	0	4	**<0.05**
Calcium channel blocker	4	3	0.67
Thyroxine	1	0	0.99
PPI	2	3	0.99

Supplementary table 2

A summary of the pharmacotherapy usage for all the participants in the study (NP+: painful diabetic neuropathy; Non-NP: painless diabetic neuropathy). Categorical data were analyzed using the Fischer's exact test (*p<0.05). Abbreviations: NSAIDS – Non-steroidal anti-inflammatory drugs; SNRI – Serotonin-noradrenergic reuptake inhibitor; ACE-I- Angiotensin converting enzyme inhibition, ARB – Angiotensin receptor blocker; B-blocker – Beta Blocker; PPI – Proton pump inhibitor

	Painful diabetic neuropathy	Painless diabetic neuropathy	P
**TCSS Total Score**	7.5 (5.3-10)	6.5 (3.3-9)	0.49
**TCSS Symptom sub-score**	2 (1.8-3)	1 (0.3-2)	**< 0.05**
**TCSS Examination sub-score**	5 (2.8-8)	5.5 (2-7.8)	0.94
**MRC Sensory Sum Score**	3.5 (1.5-6.3)	1 (0-3.5)	0.15
**IENFD (fibres/mm)**	1.1 (0.2-1.6)	1.4 (0.1-1.7)	0.40
**Sural nerve**			
**Amplitude (μV)**	3.8 (0-5.4)	4.3 (0.4-6.3)	0.74
**Conduction velocity (m/s)**	39.2 (37.6-44.6)	46.4 (40.4-50.8)	0.11
**Peroneal nerve**			
**Amplitude (mV)**	2.2 (1.2-3.4)	4.4 (2.9-6.4)	**< 0.01**
**Conduction velocity (m/s)**	38.7 (34.8-41.3)	40.9 (37.7-47.1)	0.09

Supplementary table 3

A summary of clinical scores, IENFD and electrophysiological recordings from lower limbs for all the participants in the study (NP+: painful diabetic neuropathy; Non-NP: painless diabetic neuropathy). Apart from the TCSS symptom sub-score and peroneal nerve amplitude there were no differences between the two groups. Data is shown as the median (interquartile range) and was analyzed by Mann-Whitney U Test ($p<0.05$). Significant differences between the groups are in bold. Abbreviations: TCSS – Toronto Clinical Scoring System, MRC- Medical Research Council, IENFD – Intraepidermal nerve fibre density

	Painful diabetic neuropathy	Painless diabetic neuropathy	P
**API**	4.7 (2.7-6.7)	N/A	
**DN4**	5 (4-7)	1 (0-2.8)	**< 0.01**
**painDETECT**	14 (8.5-21.5)	1.5 (0-3.5)	**< 0.01**
**BPI Pain Severity**	4.3 (2.6-6)	1.2 (0.1-2)	**< 0.01**
**NPSI Total**	14.3 (9.5-22.3)	N/A	
**NPSI Deep Spontaneous Pain**	0.5 (0-3)	N/A	
**NPSI Superficial Spontaneous Pain**	4 (0-7.3)	N/A	
**NPSI Evoked Spontaneous Pain**	1.8 (0-4)	N/A	
**NPSI Paraesthesia**	5.8 (1.9-9)	N/A	
**NPSI Paroxysmal Pain**	2.8 (0-7.1)	N/A	

Supplementary table 4

A summary of the neuropathic pain screening scores (NP+: painful diabetic neuropathy; Non-NP: painless diabetic neuropathy). Data shown as median (interquartile range) and analyzed by Mann-Whitney U Test (*p<0.05). Significant differences between the groups are in bold. Abbreviations: API – Seven-day pain diary; DN4 - Douleur Neuropathique en 4 Questions; BPI – Brief Pain Inventory; NPSI – Neuropathic Pain Symptom Inventory

	Painful diabetic neuropathy	Painless diabetic neuropathy	P
**BPI Pain Interference**	4.1 (2.4-6.1)	0.3 (0-1.4)	**< 0.01**
**PCS Rumination**	3.5 (0.8-10.3)	0 (0-6.3)	0.11
**PCS Magnification**	2.5 (1-4)	1 (0-2)	0.10
**PCS Helplessness**	4 (1.8-12)	0.5 (0-5)	**< 0.05**
**PCS Total**	11 (3.8-24)	3 (0-13.8)	**< 0.05**
**DAPOS Depression**	7.5 (5-12.5)	5.5 (5-9.5)	0.22
**DAPOS Anxiety**	3 (3-7)	3 (3-3.75)	0.26
**DAPOS Positive Outlook**	11 (7-13.3)	13 (10-15)	0.21
**PASS20 Cognitive**	6.5 (2.8-11.3)	3 (0-7.8)	0.08
**PASS20 Escape-avoidance**	4.5 (2-9.5)	2.5 (0.3-3.8)	0.06
**PASS20 Fear**	3 (0.8-7.3)	0 (0-1)	**< 0.01**
**PASS20 Physiological Anxiety**	1 (0-4)	0	**< 0.05**
**PASS Total**	14 (7.8-35)	5.5 (2.3-14)	**< 0.05**
**SAS Symptom Score**	10.5 (4.5-11.5)	4.5 (2.3-6.8)	**< 0.05**
**SAS Total Impact Score**	3 (2-4)	2 (1.3-3)	0.18
**ISI Total**	16.5 (7.5-18.5)	4 (0-7)	**< 0.01**
**SF-36 Physical Functioning**	65 (42.5-90)	85 (57.5-100)	0.12
**SF-36 Role Physical**	87.5 (0-100)	100 (100-100)	0.08
**SF-36 Bodily Pain**	46.5 (28.8-72)	78 (53.8-84)	**< 0.05**
**SF-36 General Health**	43.5 (34.3-60.5)	62 (39.5-72)	0.11
**SF-36 Vitality**	40 (20-51.3)	65 (53.8-80)	**< 0.01**
**SF-36 Social Functioning**	62.5 (46.9-90.6)	100 (100-100)	**< 0.01**
**SF-36 Role Emotional**	100 (33.3-100)	100 (33.3-100)	0.88
**SF-36 Mental Health**	64 (50-77)	84 (64-100)	**< 0.05**

Supplementary table 5

A summary of the scores for quality life measures, depression, anxiety, insomnia and autonomic symptom abnormalities for all the participants (NP+: painful diabetic neuropathy; Non-NP: painless diabetic neuropathy). All data is presented as the median (interquartile range). Data were analyzed by Mann-Whitney U test (*$p<0.05$). Significant differences between the groups are in bold. Abbreviations: BPI – Brief Pain Inventory; DAPOS - Depression, Anxiety, and Positive Outlook Scale; PCS – Pain Catastrophizing Scale; PASS-20 – short form of Pain Anxiety Symptoms Scale; SAS - Survey of Autonomic Symptoms; ISI – Insomnia Severity Index; SF-36 -Short Form 36 of the MOS Outcomes Study
