# Supplementary material for: A brain-based pain facilitation mechanism contributes to painful diabetic polyneuropathy
Source: Brain. 2018 Jan 15;141(2):357–64. doi: 10.1093/brain/awx337 (PMC5837628; doi:10.1093/brain/awx337)
Supplement: Supplementary Data [file brain-2017-01107-file007_awx337.pdf]

Supplementary materials

Sensory phenotyping

Neurological examination. A comprehensive structured upper and lower limb neurological examination was performed to detect clinical signs of a peripheral neuropathy and included an assessment of temperature, light touch and pinprick sensation, joint position proprioception, vibration perception, deep-tendon reflexes, muscle bulk, and motor power. The clinical findings were quantified with the Toronto Clinical Scoring System (TCSS) (Bril and Perkins, 2002) and MRC sensory sum score.

Nerve conduction tests and skin biopsy for intra-epidermal nerve assessment. Tests were performed with an ADVANCE system (Neurometrix, Massachusetts, USA) and used conventional reusable electrodes. Sural sensory and peroneal motor nerve conduction studies were performed (Buschbacher and Orahlow, 2006). Our protocol was in line with those recommended by the American Academy of Neurology and American Association of Electrodiagnostic Medicine (England et al., 2005). The determination of intraepidermal nerve fibre density from skin biopsy samples is a validated and sensitive diagnostic tool for the assessment of small fibre neuropathies, including diabetic neuropathy (Lauria et al., 2010). Biopsy samples were taken in accordance with the consensus document produced by the European Federation of Neurological Societies/Peripheral Nerve Society Guideline on the utilization of skin biopsy samples in the diagnosis of peripheral neuropathies (Lauria et al., 2010).

Functional magnetic resonance imaging

The following sequence parameters were used: ‘tag’ and ‘control’ images were acquired every TR=4s (in series); for each ‘tag-control’ pair, a different post-label delay time was used (PLDs = 0.25, 0.50, 0.75,1.0, 1.25, 1.50 seconds). Arterial blood was magnetically tagged using a label duration of 1.4 seconds. Other imaging parameters included: single-shot echo planar imaging (EPI), echo time (TE) = 13ms; partial Fourier = 6/8, field of view (FOV) = 220x220, matrix = 64 x 64, 24 ascending slices, slice thickness = 4.95mm; slice acquisition time = 0.0452 seconds. B0 shimming was performed over the imaging region and the

labelling plane to minimise off-resonance effects. For each participant, two calibration images were also acquired to estimate the equilibrium magnetisation of blood and to correct the pCASL data for any imbalance in the sensitivity profile of the head coil. Each calibration consisted of no labelling or background suppression, a TR = 6 seconds, and all other parameters identical to the functional pCASL scans. For the body coil calibration scan, the body coil was used for signal detection.

Supplementary results

Demographics and pharmacotherapy usage

96.2% of study participants had type 2 diabetes mellitus, in line with population prevalence, and were diagnosed with diabetes for a similar duration. Seven (50%) of the study participants with painful diabetic neuropathy and two (16.7%) of the study participants with painless diabetic neuropathy reported analgesic use (Supplementary table 2). There were no statistical differences in analgesic use between the groups.

Structured neurological examination, nerve conduction studies, IEFND and QST

All study participants had evidence of a clinical neuropathy as confirmed with the nerve conduction studies and/or IENFD results (Supplementary table 3). Only one participant did not have a skin biopsy as they declined the test. The two groups were well matched across almost all parameters.

Pain questionnaires, pain diary and body maps

Consistent with the findings in the larger PiNS cohort (Themistocleous et al., 2016), the pain experienced by the Non-NP participants was related to nonspecific lower back pain and/or musculoskeletal joint pain; confirmed with the BPI pain severity scale (Supplementary table 4). NP+ participants had significantly higher scores on the DN4, painDETECT and NPSI scales which were used to characterised the neuropathic pain symptoms experienced by the participants (Supplementary table 4). The NP+ participants exhibited elements of

psychological distress, and sleep disturbance when compared to the Non-NP cohort (Supplementary table 5).
